# A comparison of a traditional endotracheal tube versus ETView SL in endotracheal intubation during different emergency conditions: A randomized, crossover cadaver trial

**DOI:** 10.1097/MD.0000000000005170

**Published:** 2016-11-04

**Authors:** Zenon Truszewski, Paweł Krajewski, Marcin Fudalej, Jacek Smereka, Michael Frass, Oliver Robak, Bianka Nguyen, Kurt Ruetzler, Lukasz Szarpak

**Affiliations:** a Department of Emergency Medicine; b Department of Forensic Medicine, Medical University of Warsaw, Warsaw; c Department of Emergency Medical Service, Wroclaw Medical University, Wroclaw, Poland; d Department of Internal Medicine I, Medical University of Vienna, Vienna, Austria; e Outcomes Research Consortium; f Departments of Outcomes Research and General Anesthesiology, Cleveland Clinic, Cleveland, OH.

**Keywords:** cadaver, cardiopulmonary resuscitation, endotracheal intubation, laryngoscopy, paramedic

## Abstract

**Background:**

Airway management is a crucial skill essential to paramedics and personnel working in Emergency Medical Services and Emergency Departments: Lack of practice, a difficult airway, or a trauma situation may limit the ability of paramedics to perform direct laryngoscopy during cardiopulmonary resuscitation. Videoscope devices are alternatives for airway management in these situations. The ETView VivaSight SL (ETView; ETView Ltd., Misgav, Israel) is a new, single-lumen airway tube with an integrated high-resolution imaging camera. To assess if the ETView VivaSight SL can be a superior alternative to a standard endotracheal tube for intubation in an adult cadaver model, both during and without simulated CPR.

**Methods:**

ETView VivaSight SL tube was investigated via an interventional, randomized, crossover, cadaver study. A total of 52 paramedics participated in the intubation of human cadavers in three different scenarios: a normal airway at rest without concomitant chest compression (CC) (scenario A), a normal airway with uninterrupted CC (scenario B) and manual in-line stabilization (scenario C). Time and rate of success for intubation, the glottic view scale, and ease-of-use of ETView vs. sETT intubation were assessed for each emergency scenario.

**Results:**

The median time to intubation using ETView vs. sETT was compared for each of the aforementioned scenarios. For scenario A, time to first ventilation was achieved fastest for ETView, 19.5 [IQR, 16.5–22] sec, when compared to that of sETT at 21.5 [IQR, 20–25] sec (p = .013). In scenario B, the time for intubation using ETView was 21 [IQR, 18.5–24.5] sec (p < .001) and sETT was 27 [IQR, 24.5–31.5] sec. Time to first ventilation for scenario C was 23.5 [IQR, 19–25.5] sec for the ETView and 42.5 [IQR, 35–49.5] sec for sETT.

**Conclusions:**

In normal airways and situations with continuous chest compressions, the success rate for intubation of cadavers and the time to ventilation were improved with the ETView. The time to glottis view, tube insertion, and cuff block were all found to be shorter with the ETView.

**Trial Registration:**

clinicaltrials.gov Identifier: NCT02733536.

## 1 Introduction

Airway management is a crucial skill required by paramedics and other personnel working in Emergency Medical Services (EMS) and Emergency Departments (ED). According to the European Resuscitation Council (ERC) and American Heart Association's (AHA) 2015 guidelines for cardiopulmonary resuscitation (CPR), the gold standard for definitive airway management during CPR is endotracheal intubation (ETI). The guidelines also recommend limiting the interruption of chest compressions (CC) during CPR, in order to ensure better patient outcomes.^[1,2]^ It is critical that airway management happens quickly, limiting the number of interruptions in chest compressions to the least amount possible. The most common method in securing an airway is direct laryngoscopy with Miller or Macintosh blades. Numerous prehospital and simulation studies have shown that the lack of practice limits the ability of paramedics to perform direct laryngoscopy during CPR, subsequently resulting in limited effectiveness, longer hands-off times, and poorer outcomes.^[3,4,5]^ Additionally, there are other emergency situations where the glottic view can be difficult to obtain, that is, patients with neck injuries. In these cases, alternative methods of endotracheal intubation are used, such as video laryngoscopy or video tubes. The ETView VivaSight™-SL is a newly developed video tube that may be an alternative method for intubation.

**Figure 1:**
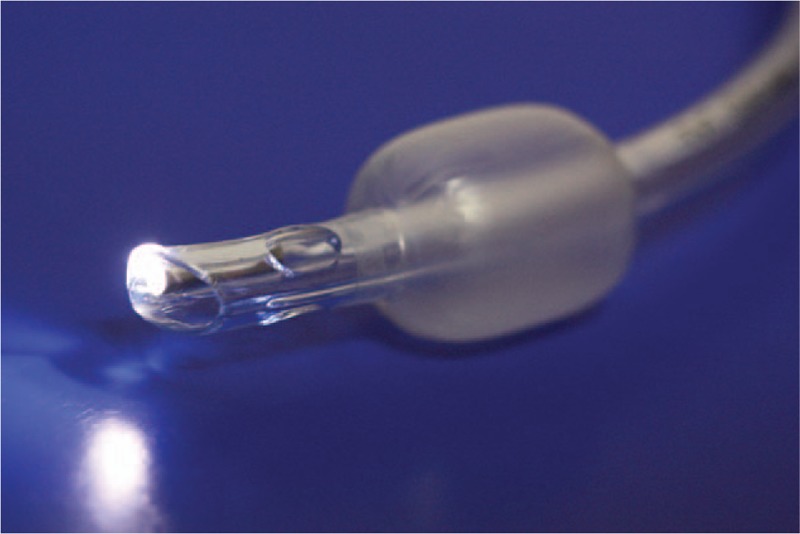
The ETView VivaSight SL video-tube.

The ETView VivaSight-SL (ETView; ETView Ltd, Misgav, Israel) is a single-lumen airway tube with an integrated high-resolution imaging camera (Fig. 1). The image is transmitted to the display in real time through a special optic fiber that can also be connected to an external monitor. This allows the clinician performing the ETI to view the entrance to the larynx and insert the endotracheal tube with direct sight. Blood or other fluids in the throat may potentially hinder the use of such devices. However, the ETView is equipped with a flushing system that allows for rapid and efficient in-situ cleaning of the camera lens. Evidence has indicated that the ETView may be suitable for tracheal intubation in various settings where standard ETI cannot be performed.^[6,7,8]^

We hypothesized that the ETView VivaSight SL could be an alternative to intubation using a standard endotracheal tube (sETT). The ETView was compared to the sETT intubation given 3 different emergency conditions using the glottis view scale, ease of use survey, along with the time and success rate of intubation

## 2 Material and methods

### 2.1 Study design and participants

This study was designed as a randomized, crossover trial. It was approved by the Institutional Review Board of the International Institute of Rescue Research and Education (Approval 24.2015.IRB) and registered at the Clinical Trials register (www.clinicaltrials.gov, identifier NCT02733536). The study was conducted between February and April of 2016. All of the experiments occurred at the Department of Forensic Medicine laboratory at the Medical University of Warsaw.

In total, 52 paramedics with <5-years of experience in Emergency Medical Services (EMS) participated in the study. The participants had not been trained on the video tube or video laryngoscopy before participating in the study. Participants had limited experience in clinical intubation (between 11 and 19 intubations). All participants were verbally informed and gave their written consent to participate in this trial.

### 2.2 Cadaver subjects

The Department of Forensic Medicine provided eligible cadavers aged anywhere from18 to 65 years at the time of death. The cadavers were screened for potential inclusion up to 72 hours after death. Exclusion criteria included fracture of the upper airway or part of the face. Ten cadavers were eligible for the study. An experienced anesthesiologist rated all bodies as CL 1 according to the criteria of the Cormack–Lehane glottic view scale.^[9]^

### 2.3 Study design

All participants completed a 30-minute training session, which included an introduction to the anatomy and physiology of the airway and techniques of endotracheal intubation using direct and video-laryngoscopy. An ETView SL with 7.0 mm internal diameter (ID) lubricated with silicon aerosol was used for video-laryngoscopy. The tube was introduced into the oral cavity using standard laryngoscope with a no.3 Macintosh blade (HEINE Optotechnik, Munich, Germany). For direct laryngoscopy, participants used a standard laryngoscope with a no.3 Macintosh blade and a standard, lubricated 7.0 ID endotracheal tube (Covidien, Mansfield, MA). All tubes used were fashioned with a hockey-stick shaped stylette and prepared by a highly experienced senior researcher in airway management. If necessary, study participants were allowed to adjust the stylette. Participants practiced intubation and placement in a classically positioned Laerdal Airway Management Trainer (Laerdal, Stavanger, Norway; 5 minutes per device). A 10 mL syringe to block the tube's cuff and an Ambu^®^ resuscitator bag (Ambu, Copenhagen, Denmark) were readily available to the participants, if necessary.

Participants performed intubations in 3 airway scenarios:

1. Scenario A: normal airway at rest without concomitant chest compressions (CC).
2. Scenario B: normal airway with continuous controlled CC was applied using the mechanical CC system LifeLine ARM (Defibtech, Guilford, CT). Chest compressions were provided in accordance to the current 2015 European Resuscitation Council (ERC) guidelines at a rate of 100 per minute to a depth of 4 to 5 cm.
3. Scenario C: Manual in-line cervical spine stabilization was performed by an instructor not involved in airway management. As the instructor stood aside the patient's chest, the lateral aspects of the patient's neck were held in the palms of his/her hand with the fingers stabilizing the mastoid process.^[10]^

**Figure 2:**
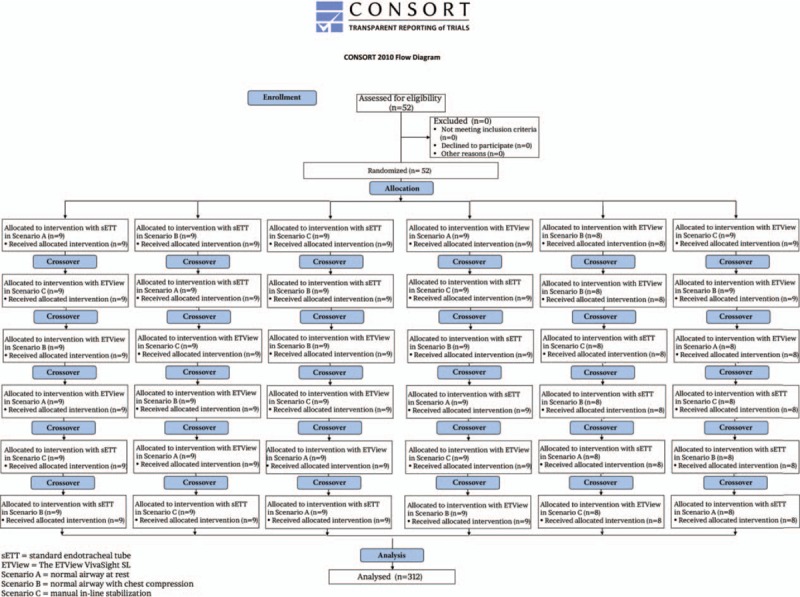
Flowchart of design and recruitment of participants according to CONSORT statement.

After the training session, a Research Randomizer program [ www.researchrandomizer.org] was used to split the volunteers into 6 groups to determine the order of laryngoscope use (Fig. 2). The first group started intubation using sETT in scenario A; the second group using sETT in scenario B; the third group, using sETT in scenario C; the fourth group, using ETView in scenario A; the fifth group, using ETView in scenario B; and the sixth group using ETView in scenario C. After completing the procedure, participants had a 10-minute break before performing the ETI attempt using the other method. Participants were not allowed to watch each other during any of the intubation attempts to avoid learning effects throughout the procedure. Participants had a maximum of 1 attempt of ETI in each condition. For ETI, the cadavers were placed on a standard autopsy table in a sniff position.

### 2.4 Outcomes

The primary outcome was time to intubation (TTI), defined as the point from first contact with the device until first successful ventilation of the lungs. Additionally, the time was also recorded from first contact with device until (a) achieving of a successful view of the glottis, (b) successful tracheal insertion of the tube, and (c) successful blocking of the tube's cuff. All measures were performed using a stopwatch.

The secondary outcome was the success rate of intubation. If the ETT was incorrectly placed or intubation lasted longer than 60 seconds, the attempt was defined as failure according to current ERC guidelines. An instructor using fiberoptic bronchoscopy confirmed correct placement of the ETT after each intubation.

Additionally, the participant was asked to rate the quality of the archived glottis view according to the Cormack–Lehane classification.^[9]^ To assess subjective opinion about ease-of-use of the intubation methods, participants were asked to rate the distinct device with a score from 1 (extremely easy) to 10 (extremely difficult). Participants were also asked which method they would prefer in real-life resuscitation.

### 2.5 Power calculation

Based on pilot data, the following assumptions were made to calculate the number of participants to be included: we proposed an alpha risk of .05, and a beta risk of 0.2. The success rate of first ETI attempt during uninterrupted CC in pilot data amounted to 95.2% versus 64.6% in the ETView and sETT, respectively. Using the *t*-test, paired, 2-sided, 32 participants were required and randomized with a 1:1 ratio.

### 2.6 Statistical analysis

The Statistica version 12.0 for Windows (StatSoft, Tulsa, OK) was used for statistical analysis. Percentages were used for qualitative variables and median with interquartile range (IQR) for quantitative variables. The occurrence of normal distribution was confirmed by the Kolmogorov–Smirnov test. Nonparametric tests were used for the data that did not have a normal distribution. All statistical tests were 2-sided. In order to compare the time needed to achieve a sufficient glottis view to that of insertion for ETT, the time to the tube's cuff was blocked and first successful ventilation was measured. The Wilcoxon test for paired observations was used to determine the statistical difference for each group. The McNemar test was used to evaluate the differences in success of intubation. The degree of pressure distribution, Cormack–Lehane grade, and VAS score were all evaluated using the Stuart–Maxwell test. A *P*-value <0.05 was considered significant.

## 3 Results

### 3.1 Study collective

In total, 52 paramedics (13 female) volunteered to participate in this study. Fifteen participants worked in an Emergency Department (ED) and 37 participants worked in Emergency Medical Service (EMS). Mean age was 27.4 (IQR, 24–30.5) years and mean work time experience was 1.7 (IQR, 0.5–2.1) years. Participant's experience with conventional ETI was 14 (IQR, 11–16) intubations.

### 3.2 Time to first ventilation

**Figure 3:**
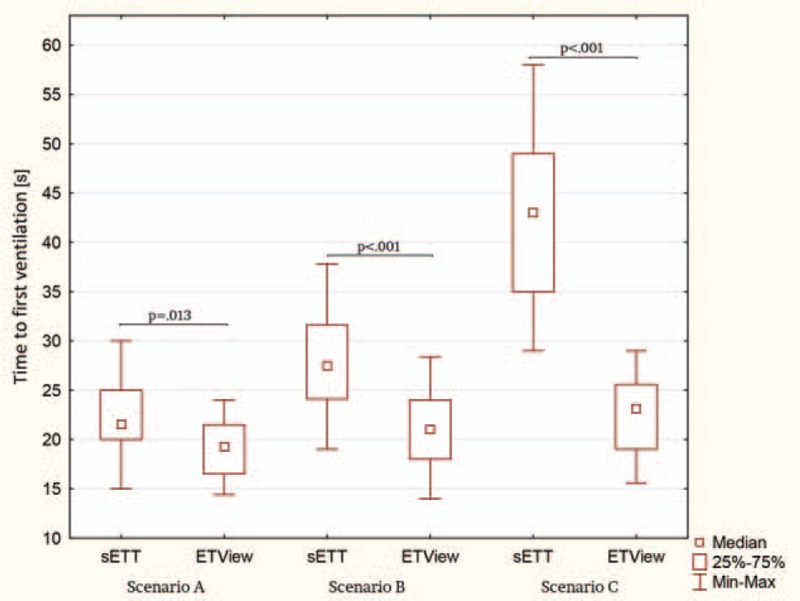
Time to first ventilation.

Results of median time to first ventilation are shown in Fig. 3. Time to first ventilation for ETView versus sETT was 19.5 (IQR, 16.5–22) verses 21.5 (IQR, 20–25)s, 21 (IQR, 18.5–24.5)s versus 27 (IQR, 24.5–31.5)s, and 23.5 (IQR, 19–25.5)s versus 42.5 (IQR, 35–49.5)s for scenarios A, B, and C appropriately. There was a statistically significant difference in the time to ventilation between ETView and sETT for scenarios A, B, and C (*P* = 0.013; *P* < 0.001; *P* < 0.001; respectively).

### 3.3 Time to glottis view, tube insertion, and cuff block

In all the 3 scenarios, the results when using the ETView were significantly better than with sETT (*P* < 0.05) for all the analyzed variables (time to glottis view, time to tube insertion, and time to cuff block). Detailed results are presented in Table 1.

**Table 1:**
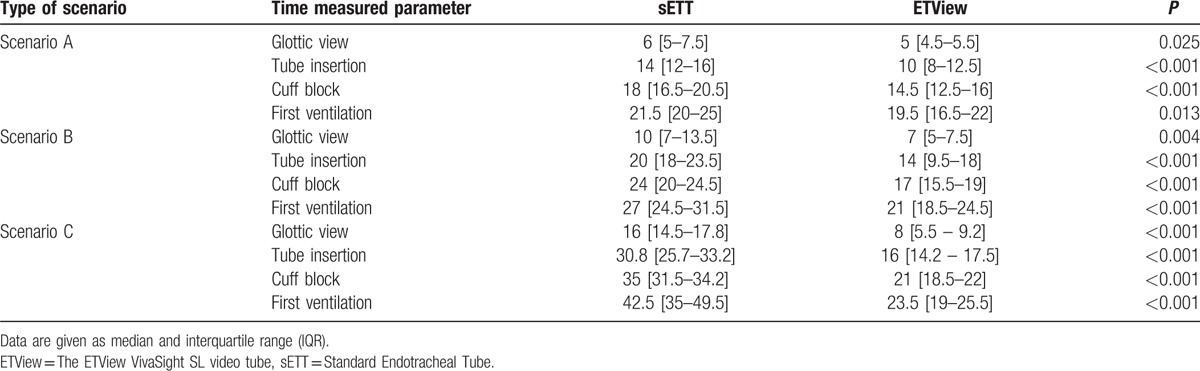
Time (s) until glottis view, tube insertion, cuff block, and first ventilation were archived.

### 3.4 Success rate

The success rate of ETI after the first attempt using sETT and ETView varied between 96.2% and 100%; 75.0% and 100%; and 61.5% and 98.1% for scenarios A, B, and C correspondingly. There was a statistically significant difference in the success rate of the intubation between sETT and ETView in scenario B (*P* < 0.001), as well as in scenario C (*P* < 0.001).

### 3.5 Quality of glottis view

The Cormack–Lehane grade of the glottis ETView for each intubation method is shown in Table 2. All participants from scenario A and B reported Cormack–Lehane grade I classification. In scenario C 51 participants grade I of Cormack–Lehane, with 1 report of a grade II.

**Table 2:**
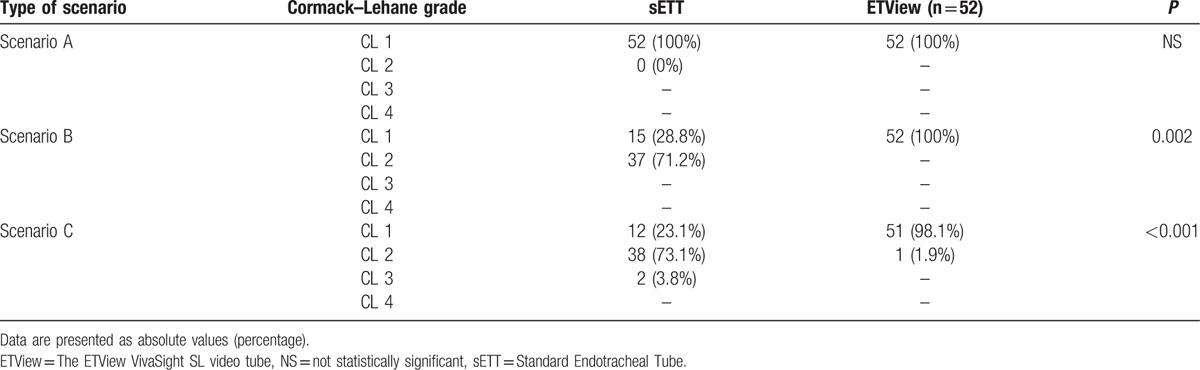
Glottis visualization, defined by Cormack and Lehane classification.

### 3.6 Subjective assessment

Participants evaluated each device via the subjective ease-of-use with which they performed the procedures. Participant's ratings of the intubation procedure using sETT and ETView varied and amounted to: 2.9 versus 2.3 points (*P* = 0.052) during scenario A; 5.5 versus 3.0 points (*P* < 0.001) during scenario B; and 6.5 versus 3.5 points (*P* < 0.001) during scenario C, respectively.

When participants were asked which intubation method they would prefer in real-life endotracheal intubation, all participants preferred the ETView to the sETT in all potential emergency scenarios.

## 4 Discussion

We demonstrate in all the 3 scenarios that the time to successful intubation, glottis view, tube insertion, and glottis view were all faster with the ETView compared to sETT. Additionally the success rate was higher with the ETView. The European Resuscitation Council guidelines for CPR state that endotracheal intubation (ETI) is the gold standard for securing airways and providing effective ventilation.^[1]^ These guidelines also stress the importance of ETI without interrupting chest compression, other than a brief pause to allow passage of the ETT. The use of alternative methods to direct laryngoscopy intubation may avoid this problem. The ETView was developed with this in mind and provides continuous real-time images of the tube position. It has already been successfully tested in manikins under such conditions.^[6]^

During the most clinically relevant scenario B (with CPR and ongoing chest compression), the median time for intubation was about 6 seconds faster using ETView. Generally, the faster time to intubation, the better, but clinical relevance using this device remains unclear. Any unnecessary interruption of chest compression may contribute to poorer patient outcomes, as interruption of cardiac perfusion is associated with increased morbidity and mortality. This study establishes that the ETView may be able to successfully establish an airway without any interruption of chest compression during CPR. Therefore, ETView may provide a positive impact on patient outcomes, minimizing hands-off time and maximizing perfusion—both important factors for resuscitation outcome.^[11,12]^

Randomized, controlled trials have shown that video laryngoscopy improves the success of first-attempt endotracheal intubation during CPR among novice physicians.^[13,14]^ In recent literature, there have been several descriptions about the safe use of the ETView. However, none of them focus on intubation utilizing the ETView during resuscitation,^[8,15]^ which is an advantageous aspect for the use of this device. In addition, the ETView has proven superior to sETT during resuscitation via simulated multitrauma studies.^[7]^

Paramedics were used as participants in this study because they are the personnel in the prehospital setting that are typically confronted with the need to intubate patients and therefore have adequate experience in airway management. In addition to the randomized, crossover design, cadavers instead of manikins were used to strengthen the legitimacy of the study, as 93% of manikins have never been tested for validity.^[16]^ Recent studies indicate that fresh human cadavers provide a realistic tool for endotracheal intubation training but results may differ largely between manikins and cadavers.^[17,18]^ Since this is a cadaver-based study, the results may be limited because the outcomes may vary when compared to that of clinical patients and will need to be verified. Use of the ETView is based on direct visualization using the integrated camera on the tip of the tube. Up to this date, no injuries have been reported from the camera and as such, this was not an investigative point for this study. However, potential decontamination with blood or spittle of the camera might affect applicability in the clinical setting. For this reason, the ETView tube is equipped with a flushing tube, but again, this facet was not examined in the present study as human cadavers do not normally have any liquid within the trachea. By nature of the study, participants could not be blinded.

More experienced paramedics may obtain better results with ETI due to daily practice and greater experience. In this study, the participants were rather young (mean age 27.4 years) and inexperienced (mean work experience 1.7 years, mean of 14 ETIs) and may have contributed to the results. A group of novice paramedics were selected to eliminate a bias associated with the experienced usage of the sETT. Another limitation is that only a single video tube device was used in this study. There are currently numerous videoscope devices available that may be used for airway management, and while we cannot provide evidence on superiority of a distinct device, this was not the central aim of the study.

## 5 Conclusions

In normal airways and situations with continuous chest compressions, the success rate for intubation of cadavers and the time to ventilation were improved with the ETView. The time to glottis view, tube insertion, and cuff block were all found to be shorter with the ETView.
